# Supplementary material for: SyNDI: synchronous network data integration framework
Source: BMC Bioinformatics. 2018 Nov 6;19:403. doi: 10.1186/s12859-018-2426-5 (PMC6219086; doi:10.1186/s12859-018-2426-5)
Supplement: Supplementary file 5 — Files of the example presented in Additional file 4. This additional file is a zip package that contains all files used in Additional file 4. Detailed descriptions of each of these files are presented in a README file included in this zip package. (GZ 30411 kb) [file 12859_2018_2426_MOESM5_ESM.gz › SyNDITools/Meme2Fimo/meme_4.9.0_4.tar.gz!/meme_4.9.0/doc/readseq.xml]

readseq


# readseq

**Usage:** `readseq [options] <in.seq> > <out.seq>`

**Description:**

Converts protein and DNA sequence data formats.

**Input:**

- A DNA or protein sequence alignment in one of the supported formats.

**Output:**

- The converted seqeuences are written to `stdout`.

**Options:**

- `-a[ll]`
  - Select all sequences.
- `-c[aselower]`
  - Change to lower case.
- `-C[ASEUPPER]`
  - Change to lower case.
- `-degap`
  - Remove gap symbols.
- `-i[tem=2,3,4]`
  - Select item number(s) from several.
- `-o[output=]out.seq`
  - Redirect output.
- `-p[ipe]`
  - Pipe (command line, <stdin, >stdout).
- `-r[everse]`
  - Change to reverse complement.
- `-v[erbose]`
  - Verbose progress.
- `-f[ormat=]#`
  - Format number for output, or
- `-f[ormat=]Name`
  - Format name for output:

  |  |  |
  | --- | --- |
  | 1. IG/Stanford 2. GenBank/GB 3. NBRF 4. EMBL 5. GCG 6. DNAStrider 7. Fitch 8. Pearson/Fasta 9. Zuker (in-only) | 1. Olsen (in-only) 2. Phylip3.2 3. Phylip 4. Plain/Raw 5. PIR/CODATA 6. MSF 7. ASN.1 8. PAUP/NEXUS 9. Pretty (out-only) |

Pretty format options:

- `-wid[th]=#`
  - Sequence line width.
- `-tab=#`
  - Left indent.
- `-col[space]=#`
  - Column space within sequences line on output.
- `-gap[count]`
  - Count gap characters in sequence numbers.
- `-nameleft, -nameright[=#]`
  - Name on left/right side `[=max width]`.
- `-nametop`
  - Name at top/bottom.
- `-numleft, -numright`
  - Name on left/right side `[=max width]`.
- `-numtop, -numbot[=#]`
  - Index on top/bottom.
- `-match[=.]`
  - Use match base for 2 .. n species.
- `-interline[=#]`
  - Blank line(s) between sequence blocks.

**Warning messages:**

- None

**Bugs:**
None known.

**Author:**
readseq was written by D. G. Gilbert
